# High Rate of Cytomegalovirus Detection in Cholestatic Preterm Infants

**DOI:** 10.3389/fped.2021.754941

**Published:** 2021-11-24

**Authors:** Jonas Teng, Anne Elwin, Soley Omarsdottir, Giulia Aquilano, Mireille Vanpee, Antal Nemeth, Afsar Rahbar, Kajsa Bohlin, Björn Fischler, Cecilia Söderberg-Nauclér

**Affiliations:** ^1^Division of Pediatrics, Department of Clinical Science, Intervention and Technology (CLINTEC), Karolinska Institutet, Stockholm, Sweden; ^2^Department of Pediatrics, Södertälje Hospital, Stockholm, Sweden; ^3^Department of Neonatology, Karolinska University Hospital, Stockholm, Sweden; ^4^Department of Medicine, Microbial Pathogenesis Unit, BioClinicum, Karolinska Institutet, Stockholm, Sweden; ^5^Department of Women's and Children's Health, Karolinska Institutet, Stockholm, Sweden; ^6^Department of Pediatrics, Karolinska University Hospital, Stockholm, Sweden; ^7^Department of Neurology, Karolinska University Hospital, Stockholm, Sweden

**Keywords:** cytomegalovirus, liver diseases, cholestasis, preterm infants, neonatal intensive care (unit), neonatal hyperbilirubinemia, neonatal jaundice

## Abstract

**Objectives:** To evaluate the prevalence of cytomegalovirus (CMV) infection in preterm infants with cholestasis.

**Study design:** Preterm infants (<37 weeks gestational age) with cholestasis were tested for CMV DNA using Taqman PCR in blood cells from sedimented whole blood, plasma, and urine. Infants were regarded as positive for CMV if any sample was tested positive. Their mothers were tested for CMV serostatus simultaneously. A control group of non-cholestatic preterm infants, and their mothers, were tested at a similar age.

**Results:** A total of 69 preterm infants with a median gestational age of 26 weeks and 5 days were included, 45 cholestatic and 24 non-cholestatic. Of the cholestatic infants, 31/45 (69%) were CMV positive vs. 3/24 (13%) of the non-cholestatic infants (*p* < 0.001). Cholestatic infants were equally preterm as the non-cholestatic ones, but were more severely ill. After adjusting for the risk factors necrotizing enterocolitis, prolonged parenteral nutrition, and gestational age, being CMV positive remained significantly associated with cholestasis in a multivariable logistic regression model. Characteristics of CMV-positive and -negative cholestatic infants showed differences only for necrotizing enterocolitis, occurring in 55% (17/31) of CMV positive vs. 21% (3/14) of CMV negative (*p* = 0.054), and mortality. Eight cholestatic CMV-positive infants died (26%) vs. none of the CMV-negative infants (*p* = 0.044).

**Conclusions:** CMV DNA was detected in two out of three cholestatic preterm infants, by far more often than in the non-cholestatic control group. Cholestasis with simultaneous detection of CMV DNA may be associated with increased mortality.

## Introduction

Human cytomegalovirus (CMV) infection is among the most common viral infections in neonates (1). Early in life, CMV may infect the fetus during pregnancy, the child at labor, or postnatally. The outcome of a congenital infection may vary from asymptomatic to multiple severe symptoms and then present with low birth weight, microcephaly, liver disease with jaundice, retinitis, hearing and vision loss, and developmental and motor delays (2, 3).

In a meta-analysis, the prevalence of congenital CMV infection (cCMV) was estimated to 0.64%, with higher prevalence in low-income countries, and 11% were estimated to be symptomatic (3). Kadambari et al. found an increase in hospital diagnoses of cCMV infections in England over the last decades, presumably due to improved diagnostics. They also found that a substantial burden of disease lay among the preterm infants in neonatal wards (4).

Postnatally acquired CMV infection (pCMV) in neonates is less well-studied. It occurs mainly through breast milk, but also through blood products or *via* horizontal transmission (5, 6). It is considered to be less often symptomatic in term infants than in preterm infants. In preterm infants, a stronger association to symptomatic disease and a more unfavorable outcome has been described (7). CMV has also been detected in surgical intestinal specimens (8) in preterm infants with necrotizing enterocolitis (NEC), but it is unclear if and how the virus is involved in the pathogenesis of this disease.

CMV is known to affect the liver, both in congenitally infected infants and when infected later in life. The rates of hepatic involvement in cCMV differ between studies. Dreher et al. reported in 2014 that >50% of neonates with symptomatic cCMV had conjugated hyperbilirubinemia (9). In 2015, Bilavsky et al. found that 6.6% of the symptomatic cCMV-infected neonates in their study had hepatic involvement, and 3% of them were cholestatic (10). Cholestasis in term infants is a rare condition of different causes where biliary atresia is the most common (11). CMV has been associated with biliary atresia and other cholestatic conditions in infancy (12–16). In preterm infants, cholestasis is far more common than in term infants, and the condition is related to prolonged parenteral nutrition, often due to feeding intolerance secondary to bowel disease such as NEC with subsequent intestinal failure (17–19), but the potential importance of CMV in this condition is not well-investigated. Neuberger et al. reported conjugated hyperbilirubinemia in 3/40 of infants with birth weight <1,000 g or gestational age <30 weeks postnatally, infected with CMV through breast milk, vs. 1/40 in matched controls, a non-significant difference (20). The aim of this study was to evaluate the prevalence of CMV infection in preterm infants with and without cholestasis.

## Materials and Methods

The study was performed prospectively at the neonatal units at Karolinska University Hospital, Stockholm, Sweden. Inclusion criteria were preterm infants (gestational age < 37 weeks) admitted to the hospital's neonatal units from March 2008 to July 2010 who subsequently developed cholestasis during the neonatal period. Cholestasis was defined as conjugated serum bilirubin ≥30 μmol/L, and more than 20% of the total serum bilirubin level. A total of 45 cholestatic preterm infants were included.

A control group of 24 preterm infants without cholestasis born from January 2015 to September 2017 was recruited from the same neonatal units and was matched for gestational age.

Blood and urine samples were collected from all infants as well as blood samples from the mothers to analyze the presence of CMV deoxyribonucleic acid (DNA) and CMV immunoglobulin G (IgG) and immunoglobulin M (IgM) in serum. In the cholestatic group, these samples were collected as soon as possible after cholestasis was detected. Samples from the control group were collected at 3–6 weeks postnatal age. This age for sampling was chosen not to precede the average age at onset of cholestasis in different populations of preterm newborn infants described (21–23). The blood samples for the study were always collected at the same point in time when blood tests for clinical reasons were performed, to avoid unnecessary pain for the infant. A maximum amount of 0.5 ml of blood was drawn to minimize the negative effects on hemodynamics and iron status of the infant. After sedimentation of cells, the plasma was removed and the rest of the whole blood sample was analyzed separately. The urine sample was collected from a consecutive urine portion after blood sampling. Maternal testing was done on the same day as infant blood sampling.

### Serology

Maternal plasma samples were tested for CMV-specific IgG and IgM using Enzygnost Anti-CMV/IgG and Enzygnost Anti-CMV/IgM kits according to the manufacturer's instructions; a cutoff value of OD: 0.2 was considered positive (Siemens Healthcare Diagnostics Products, Germany).

### DNA Extraction and Taqman PCR Assay (qPCR)

DNA was extracted from infant sedimented whole blood and plasma samples, respectively, with QIAamp DNA Blood Mini Kit (Qiagen, US), and plasma and urine samples with QIAamp DNA Kit (Qiagen) according to the manufacturer's instructions. Extracted DNA was quantified using Nanodrop 2000 (Thermo Fisher Scientific). Approximately 100 ng of DNA per sample was amplified with TaqMan Fast Universal PCR Master Mix (Life Technologies) using specific CMV immediate early DNA (CMV-IE DNA) primers: Forward Primer Seq GTGACCCATGTGCTTATGACTCTAT, Reverse Primer Seq CTCAACATAGTCTGCAGGAACGT, and IEDNA-probes Reporter TTGGTCACGGGTGTCTC Quencher (custom-made TaqMan assays, Applied Biosystems) (24).

RNase P (as housekeeping gene, assay ID, 4316844, Life Technologies, Thermo Fisher Scientific Corporation) was used for normalization. The polymerase chain reaction (PCR) was performed using a 7900HT Fast Real-Time PCR system (Applied Biosystems, USA). Positive and negative controls were DNA from CMV strain-AD169 infected and uninfected fibroblasts (MRC5, ATCC). The ΔCT method was used for calculation of cycle threshold (CT) values.

The CT for the quantitative PCR was set at 35 for a positive test. Positive PCR results were recorded for each sample: sedimented whole blood, plasma, and urine. Infants who were positive in any test were categorized as CMV positive and all others were considered CMV negative. If there were missing data for any CMV PCR sample, the result was assumed to be negative to avoid overestimating the results.

Clinical data were collected from hospital medical records and the Swedish neonatal quality register (SNQ). Small for gestational age was defined as birth weight <2 standard deviations below the mean. Sepsis was defined as blood culture positive and patent ductus arteriosus as requiring any treatment (pharmacological and/or surgical). Intraventricular hemorrhage (IVH) was reported according to grade (25), NEC according to Bell's stage ≥2 (26), and retinopathy of prematurity (ROP) according to grade (27). Bronchopulmonary dysplasia (BPD) was defined as need for oxygen at 36 weeks corrected gestational age (28). Age of cholestasis onset was set to the first day of age fulfilling the definition of cholestasis described earlier. Mortality reported was death before discharge from neonatal care.

All clinical data were collected before registering the results of the CMV PCR tests in the database in order to blind the researchers as much as possible and reduce the risk for bias. Comparisons were made between the cholestatic infants and the non-cholestatic reference group, and within the cholestasis group between the CMV-positive and CMV-negative infants.

### Statistical Analysis

SPSS for Mac version 26.0 (IBM, Armonk, NY, USA) was used for data management and statistical analyses. Fisher exact test was used as significance test for proportions. When medians and interquartile range was reported for continuous variables, Mann–Whitney *U*-test was used. Logistic regression was used for calculating odds ratios for infant CMV status and known risk factors for cholestasis, and adjusted odds ratios were calculated in a multivariable model. Significance level was always set at 5%.

## Results

### Cholestatic vs. Non-cholestatic Preterm Infants

A total of 69 infants were included in the study, with a median gestational age of 26 weeks and 5 days and birth weight 0.845 kg. Nine infants in the cholestasis group and four in the non-cholestasis group were from duplex pregnancies. The cholestasis group (*n* = 45) and the non-cholestatic reference group (*n* = 24) were similar regarding baseline characteristics as shown in Table 1. The median gestational age was 26 5/7 weeks (range 23 1/7–35 2/7 weeks) for cholestatic infants, and 26 4/7 weeks (range 23 3/7–33 4/7 weeks) for the non-cholestatic controls. The proportion of very preterm infants (<32 weeks) was 37/45 (82.2%) among the cholestatic and 22/24 (91.7%) among the non-cholestatic infants (*p* = 0.48).

**Table 1:** Characteristics of cholestatic and non-cholestatic preterm infants.

	**Cholestatic (*n* = 45)^a^**	**Non-cholestatic (*n* = 24)^a^**	***p*** **^b^**
**Mother and pregnancy**			
Maternal age, years	31 (27, 35)	33 (29.5, 35)	0.32
Singleton	36/45 (80)	20/24 (83)	1.00
Caesarian section	33/45 (73)	16/24 (67)	0.59
**Neonatal characteristics**			
Gestational age, week^+days^	26^+5^ (25^+4^, 29^+1^)	26^+4^ (24^+6^, 27^+3^)	0.31
Birth weight, g	963 (738, 1,194), *n* = 44	777 (606, 1,002)	0.053
Small for gestational age	15/44 (34)	8/24 (33)	1.00
Female gender	21/45 (47)	10/24 (42)	0.80
**Neonatal course**			
Mechanical ventilation, days	23 (12, 36)	5.5 (0, 11)	<0.0001^*^
Erythrocyte transfusions	14 (11, 21)	6 (3.5, 8)	<0.000001^*^
Lowest thrombocyte count, 10^9^/L	43 (15, 77)	146 (68, 188)	<0.00001^*^
Sepsis	28/45 (62)	8/24 (33)	0.026^*^
Patent ductus arteriosus	29/45 (64)	9/24 (38)	0.043^*^
IVH, ≥grade 3	5/45 (11)	1/24 (4)	0.657
NEC, ≥stage 2	20/45 (44)	1/24 (4)	<0.001^*^
Stoma	11/45 (24)	0/24 (0)	<0.01^*^
**Nutrition and growth**			
Age regaining birth weight, days	7 (5, 11.5), *n* = 35	7 (6, 10)	0.94
Parenteral nutrition, ≥2 weeks	42/45 (93)	11/24 (46)	<0.0001^*^
Any maternal breast milk	36/45 (87)	23/24 (96)	0.408
Any donor breast milk	32/44 (73)	20/21 (95)	0.046^*^
**Liver function tests**			
ALT peak, μkat/L^c^	1.45 (0.79, 2.27), *n* = 44	0.15 (0.11, 0.20), *n* = 16	<0.0000001^*^
AST peak, μkat/L^c^	2.53 (1.51, 4.10), *n* = 44	0.46 (0.35, 0.64), *n* = 15	<0.0000001^*^
γ-GT peak, μkat/L^c^	2.80 (2.00, 4.80), *n* = 41	0.57 (0.52, 1.1), *n* = 9	<0.0001^*^
**Outcomes**			
BPD	24/40 (60)	8/24 (33)	0.070
ROP ≥ stage 3	12/42 (29)	4/24 (17)	0.375
Mortality	8/45 (18)	1/24 (4)	0.15

*IVH, intraventricular hemorrhage; NEC, necrotizing enterocolitis; ALT, alanine aminotransferase; AST, aspartate aminotransferase; γ-GT, gamma glutamyl transpeptidase; BPD, bronchopulmonary dysplasia; ROP, retinopathy of prematurity*.

a *n/N (%) for proportions, median ([IQR:] 25th percentile, 75th percentile) for continuous variables*.

b *Fisher exact test for proportions, Mann–Whitney U-test for continuous variables*.

c *1 μkat/L ≈ 60 U/L. Upper reference: ALT < 0.85 μkat/L, AST < 1.4 μkat/L, γ-GT < 0.27 μkat/L*.

* *Statistical significance*.

The cholestatic infants had a more complicated neonatal course with a greater need for mechanical ventilation and longer time with parenteral nutrition, which was also reflected in more cases of NEC and treatment-requiring patent ductus arteriosus (Table 1).

The maternal CMV serostatus did not differ between the cholestatic and the non-cholestatic infants, as shown in Table 2, and was consistent with the estimated seroprevalence in the population (27). In 11 cases for the cholestatic group and 8 cases in the non-cholestatic group, a blood test was not obtained from the mother. Three of 34 (8.8%) mothers to the cholestatic infants were CMV IgM positive, but none of 24 mothers of the non-cholestatic infants.

**Table 2:** CMV status in cholestatic and non-cholestatic infants.

	**Cholestatic (*n* = 45)^a^**	**Non-cholestatic (*n* = 24)^a^**	***p*** **^b^**
**Maternal CMV serostatus**			
IgG +, IgM −	23/34 (68)	11/16 (69)	1.00
IgG +, IgM +	2/34 (6)	0/16 (0)	1.00
IgG −, IgM +	1/34 (3)	0/16 (0)	1.00
IgG −, IgM −	8/34 (24)	5/16 (31)	0.73
**Infant CMV DNA PCR**			
Sedimented whole blood +	12/45 (27)	3/24 (13)	0.23
Urine +	6/30 (20)	0/21 (0)	0.036^*^
Plasma +	27/45 (60)	0/18 (0)	0.000007^*^
Positive in any sample	31/45 (69)	3/24 (13)	<0.000009^*^
Age at blood sampling, days	32 (19, 41), *n* = 41	29 (27, 38), *n* = 22	0.53

*CMV, cytomegalovirus; IgG, immunoglobulin G; IgM, immunoglobulin M*.

a *n/N (%) for proportions, median ([IQR:] 25th percentile, 75th percentile) for continuous variables*.

b *Fisher exact test for proportions, Mann–Whitney U-test for continuous variables*.

* *Statistical significance*.

**Figure 1:**
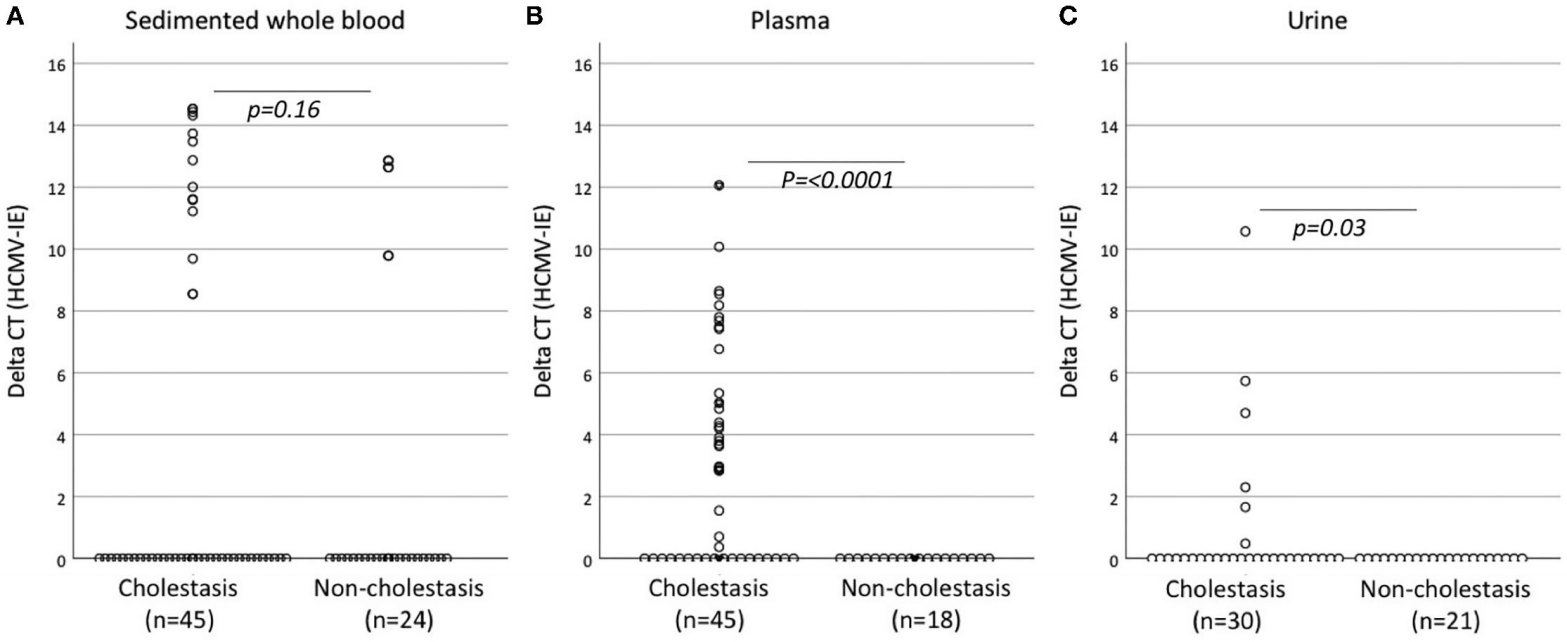
Delta CT levels for CMV-IE DNA in samples from sedimented whole blood **(A)**, plasma **(B)**, and urine **(C)** from Taqman PCR assay. Mann–Whitney *U*-test was used as significance test for distributions. CT, cycle threshold; CMV-IE DNA, cytomegalovirus immediate early DNA.

The prevalence of any positive CMV PCR test was significantly higher among the cholestatic infants than in the reference group, 68.9% vs. 12.5% (Table 2). The most profound differences were seen in the plasma and urine samples, although there were some missing data regarding these samples, as shown in Figure 1. Figure 1 also shows the delta CT values for CMV-IE from the qPCR analysis. Missing samples from the infants were mainly due to difficulties in obtaining urine samples or due to the small amount of blood drawn from the infants, not always allowing for both sedimented blood cell and plasma PCR analysis. The distribution of positive CMV-IE DNA samples from different sample locations is shown in Supplementary Figure 1. One CMV-positive cholestatic infant was treated with ganciclovir. In this patient, at the discretion of the clinicians, CMV DNA was analyzed and found negative by PCR on stored Guthrie card used for neonatal screening program (29). A congenital infection was considered to be ruled out and peri- or postnatal infection was assumed.

ALT, AST, and GT levels were significantly higher in cholestatic infants than in non-cholestatic controls (Table 1). Peak conjugated bilirubin levels correlated strongly to peak ALT (*r* = 0.76, *p* < 0.001) and peak AST (*r* = 0.82, *p* < 0.001) but not to γ-GT (*r* = 0.26, *p* = 0.075) using Spearman's rank correlation. None of the cholestatic infants were diagnosed with biliary atresia. The vast majority was diagnosed as cholestasis due to prematurity and need for parenteral nutrition. In addition, one case of hypothyroidism was found and treated among the cholestatic infants, but no other infant was diagnosed with any other endocrinological or metabolic disorders. The levels of α-1-antitrypsin were determined in 10 of the cholestatic infants, and all had normal levels.

### CMV-Positive vs. CMV-Negative Cholestatic Infants

In Table 3, the CMV-positive cholestatic infants are compared to the CMV-negative cholestatic infants. The baseline characteristics did not differ between groups. The course of cholestasis showed no difference between CMV-positive and CMV-negative infants, neither did severity of cholestasis, measured as peak conjugated bilirubin. Of the CMV-positive cholestatic infants, 17/31 (55%) had NEC as compared to 3/14 (21%) of the CMV-negative infants, a difference just above the significance level (*p* = 0.054). Notably, all eight (17.7%) cholestatic infants who died were CMV positive, while none of the CMV-negative infants died (*p* = 0.044, Table 3). Among the CMV-positive cholestatic infants, 8/31 (26%) died. Seven of the deceased infants had ongoing cholestasis at the time of death. There was no difference in the incidence of BPD or ROP between CMV-positive and CMV-negative infants (Table 3).

**Table 3:** CMV-positive vs. CMV-negative cholestatic infants.

	**CMV PCR in any sample (sedimented whole blood, plasma or urine)**		***p*** **^b^**
	**Positive (*n* = 31)^a^**	**Negative (*n* = 14)^a^**	
**Mother and pregnancy**			
Maternal age	31 (27, 35)	32.5 (27, 35)	0.74
Preeclampsia	6/31 (19)	0/14 (0)	0.16
Chorioamnionitis	6/31 (19)	3/14 (21)	1.00
Steroids, antenatal	27/31 (87)	12/14 (86)	1.00
Cesarean delivery	23/31 (74)	10/14 (71)	1.00
**Neonatal characteristics**			
Gestational age, week^+day^	27^+0^ (26^+0^, 29^+2^)	26^+1^ (25^+1^, 26^+6^)	0.19
Birth weight, g	997 (735, 1,235), *n* = 30	903 (748, 1,070)	0.61
Female gender	14/31 (45)	7/14 (50)	1.00
Small for gestational age	10/30 (33)	5/14 (36)	1.00
**Cholestasis**			
Age at cholestasis onset, days	18 (7.5, 23)	20 (8, 30)	0.55
Duration cholestasis, CB <30 μmol/L	67 (28, 84), *n* = 23	77 (71, 99)	0.13
TB peak before cholestasis onset, μmol/L	164 (140, 208), *n* = 29	170 (120, 200)	0.80
TB peak during cholestasis, μmol/L	168 (144, 271)	150 (106, 241)	0.35
CB peak, μmol/L	117 (70, 195)	113 (75, 164)	0.85
Age at peak CB, days	40 (24, 61)	49 (32, 65)	0.57
ALT peak, μkat/L^c^	1.05 (0.76, 1.97)	1.94 (1.38, 2.54), *n* = 13	0.21
AST peak, μkat/L^c^	2.30 (1.49, 3.29)	3.96 (1.98, 4,78)	0.27
γ-GT peak, μkat/L^c^	3.05 (2.00, 4.95), *n* = 28	2.60 (1.70, 3.80), *n* = 13	0.31
INR peak	2.3 (1.8, 2.7)	2.3 (1.9, 2.9)	0.66
Lowest thrombocyte count, 10^9^/L	34 (13, 84.5)	52.5 (23, 74)	0.48
Vitamin K treatment	25/30 (83)	14/14 (100)	0.16
Ursodeoxycholic acid treatment	26/29 (90)	14/14 (100)	0.54
**Maternal CMV serostatus**			
IgG +, IgM –	15/22 (68)	8/12 (67)	1.00
IgG +, IgM +	1/22 (5)	1/12 (8)	1.00
IgG −, IgM +	0/22 (0)	1/12 (8)	0.35
IgG –, IgM –	6/22 (27)	2/12 (17)	0.68
**Neonatal course**			
Mechanical ventilation, days	24 (12, 40)	23 (16, 33)	0.94
Lowest thrombocyte count, × 10^9^/L	34 (13, 85)	53 (23, 74)	0.48
Sepsis	21/31 (67)	7/14 (50)	0.33
Blood transfusions	14 (9, 21)	17 (13, 21)	0.42
Necrotizing enterocolitis	17/31 (55)	3/14 (21)	0.054
Stoma	9/31 (29)	2/14 (14)	0.46
Patent ductus arteriosus	19/31 (61)	10/14 (71)	0.38
IVH, ≥grade 3	4/31 (13)	1/14 (7)	1.00
**Nutrition and growth**			
Any maternal breast milk	26/31 (84)	13/14 (93)	0.65
Maternal breast milk before cholestasis	20/29 (69)	8/14 (57)	0.51
Any donor breast milk	22/30 (73)	10/14 (71)	1.00
Donor breast milk before cholestasis	19/29 (66)	10/14 (71)	1.00
Parenteral nutrition, ≥2 weeks	29/31 (94)	13/14 (93)	0.48
Clinoleic (Baxter) at any time	14/31 (45)	8/14 (57)	0.53
Intralipid (Fresenius Kabi) at any time	20/31 (65)	8/14 (57)	0.74
Omegaven (Fresenius Kabi) at any time	13/31 (42)	6/14 (43)	1.00
**Outcomes**			
ROP, ≥grade 3	8/28 (29)	4/14 (29)	1.00
BPD, grade 2 or higher	15/26 (58)	9/14 (64)	0.75
Mortality	8/31 (26)	0/14 (0)	0.044^*^
Hearing disability in childhood	3/13 (23)	1/10 (10)	0.60

*CMV, cytomegalovirus; PCR, polymerase chain reaction; CB, conjugated bilirubin; TB, total bilirubin; ALT, alanine aminotransferase; AST, aspartate aminotransferase; γ-GT, gamma glutamyl transpeptidase; INR, international normalized ratio; IgG, immunoglobulin G; IgM, immunoglobulin M; IVH, intraventricular hemorrhage; ROP, retinopathy of prematurity; BPD, bronchopulmonary dysplasia*.

a *n/N (%) for proportions, median ([IQR:] 25th percentile, 75th percentile) for continuous variables*.

b *Fisher exact test for proportions, Mann–Whitney U-test for continuous variables*.

c *1 μkat/L ≈ 60 U/L. Upper reference: ALT < 0.85 μkat/L, AST < 1.4 μkat/L, γ-GT < 0.27 μkat/L*.

* *Statistical significance*.

### Risk Factors for Cholestasis

Crude odds ratios for CMV status and the known risk factors of prolonged parenteral nutrition (≥2 weeks) and NEC (≥Bell's stage 2), as well as the odds ratio for gestational age are presented in Table 4. Matching for GA was attempted but incomplete and it was therefore included as a factor to adjust for. The significant crude association between being CMV DNA positive and having a diagnosis of cholestasis remained after adjusting for the variables described above (Table 4). Models including sepsis (alone or in combination with NEC) instead of NEC alone therefore yielded similar results (data not shown).

**Table 4:** CMV and risk factors for cholestasis, multivariate analysis.

	**Crude OR**	**95% CI**	***p***	**Adjusted OR**	**95% CI**	***p***
CMV DNA positive^a^	15.5	4.0, 60.7	0.000082*	5.5	1.1, 26.6	0.036*
PN ≥2 weeks^b^	16.6	4.0, 68.5	0.00011*	29.6	2.3, 377.4	0.0091*
Necrotizing enterocolitis^c^	18.4	2.3, 148.3	0.0062*	6.4	0.66, 61.7	0.11
Gestational age^d^	1.14	0.94, 1.38	0.18	1.41	0.98, 2.02	0.065

*Multivariable logistic regression model for positive CMV DNA adjusted for known risk factors for cholestasis. Nagelkerke R square for model: 0.61*.

a *CMV DNA positive in sedimented whole blood, plasma or urine. Reference: CMV DNA negative in all locations*.

b *Reference: PN <2 weeks*.

c *Reference: No diagnosis of necrotizing enterocolitis ≥Bell's stage 2*.

d *Per additional day of gestational age*.

*OR, odds ratio; CI, confidence interval; CMV, cytomegalovirus; DNA, deoxyribonucleic acid; PN, parenteral nutrition*.

## Discussion

The aim of this study was to examine the prevalence of CMV in cholestatic preterm infants. Our main finding was that CMV-IE DNA was found in more than two-thirds of preterm infants with cholestasis in the neonatal wards.

The prevalence of CMV-IE DNA in sedimented whole blood, plasma, or urine was significantly higher in cholestatic preterm infants than in non-cholestatic preterm control infants with similar gestational age and postnatal age. The two groups differed significantly in other aspects as well. For example, cholestatic infants were sicker with more ventilatory support and sepsis and also had higher rates of known risk factors for cholestasis such as NEC with intestinal failure and prolonged parenteral nutrition. When adjusting for these risk factors, and for gestational age, being CMV positive remained associated with cholestasis.

The seroprevalence of maternal CMV IgG did not differ between the groups, and neither did the rate of being fed maternal breast milk. The IgM prevalence trended higher in mothers of cholestatic infants than expected among healthy individuals who rarely are CMV IgM positive (30), but this was not statistically significant possibly due to low number of individuals in each group.

As the prevalence of CMV in cholestatic infants was higher than in non-cholestatic controls, the question arises; how did the cholestatic infants become infected with CMV? It is possible that some of them had a congenital infection or were infected at birth. The infection could also be transferred by breast milk, but both groups had a similar rate of being fed breast milk. The cholestatic infants were sicker and required more mechanical ventilation, parenteral nutrition, and transfusion of blood products, wherefore it cannot be excluded that the infection could be iatrogenic. CMV is present in a latent state in monocytes in CMV-positive blood donors. These monocytes are eliminated by filters prior to transfusion, taking away most, but not all the cells that potentially could transfer latent CMV. CMV is reactivated from latency in macrophages upon inflammatory stimuli (31, 32). If CMV-positive monocytes are still present in erythrocyte concentrates given to patients, the virus may reactivate from latency when monocytes differentiate into macrophages, especially in those with high ongoing inflammatory activity such as patients with sepsis or NEC. In the present study, we do not have information regarding timing of transfusions, i.e., before or after the patients became cholestatic or developed NEC. However, if the virus reaches the liver, it could participate in the pathogenesis of cholestasis. Likewise, if entering into inflamed tissues such as in the bowel of NEC patients, the virus could potentially worsen the inflammation and affect the patient's prognosis. In support of this hypothesis, CMV has been detected in a high proportion of intestinal surgical specimens in preterm infants with NEC (33). We therefore speculate that CMV replication taking place in the intestines of preterm infants may be a source of CMV DNA in plasma. It is also possible that the liver in cholestatic infants has infected foci releasing virus into the circulation.

When comparing CMV-positive and negative cholestatic infants, the groups were very similar regarding the neonatal course and morbidities except for a trend of more NEC among the CMV-positive cholestatic infants.

Of concern is that among cholestatic infants who died, all were CMV positive. Mortality before discharge was 26% among CMV-positive cholestatic infants, while none of the CMV-negative cholestatic infants died. Mortality figures can be hard to compare between different groups, but to put in perspective, this figure could be compared to the neonatal mortality (death on day 0–27 of life in live born infants) in preterm infants (<37 weeks) in 2009 and 2016, which was virtually unchanged in Sweden at a level around 1.8% during the time period 2008–2017 (official data from the National Board of Health and Welfare in Sweden, Socialstyrelsen.se) or to the 1-year mortality of 8.9% in infants with gestational age of 26 full weeks at birth in Sweden in 2020 (official statistics from the Swedish Neonatal Quality Register, snq.se). If CMV actually contributes to higher mortality, it would be important to evaluate if antiviral treatment can prevent the potentially negative effects of the virus on the patient's condition.

In order to define patients potentially in need of such antiviral treatment, systematic detection of CMV would be needed. Since we observed that CMV tests could be positive in one sample location and negative in another in the same infant, it may be necessary to test for CMV in samples obtained from several locations to detect viral DNA in clinical practice.

The strength of this study is the prospective approach, the sampling from several locations, and the inclusion of a control group without cholestasis. To our knowledge, this type of prospective study has not been performed earlier in this specific patient population. This study has several weaknesses. One weakness is that there were some missing data due to incomplete sampling, but assuming missing samples to be negative for CMV reduces the risk of over-interpretation of the results. The fact that the control group was enrolled at a later time point than the cholestatic cases is another weakness. The cases and controls had similar gestational ages and were sampled at similar ages, and the same laboratory facilities used the same method for PCR analysis, but the fact that they were included in different time periods increases the risk of introducing unknown bias. We aimed at including 45 control infants to match the 45 cholestatic infants, but had to terminate inclusion due to difficulties in finding enough non-cholestatic preterm infants within reasonable time that fulfilled the criteria for inclusion. Another weakness is that the design of the study prevents us from distinguishing between congenital and postnatal CMV infection. To address this, a cohort study with early and repeated tests should be performed.

The design of the study prevents us from drawing firm conclusions on causality of CMV infection and cholestasis. We cannot conclude whether the CMV detected in the infants was transmitted congenitally or postnatally, even though we assume that the great majority of our cohort was postnatally exposed. However, when considering the very clear difference in CMV rate between cholestatic and non-cholestatic preterm infants found in this study, we would argue that further studies are warranted on the prevalence and importance of CMV in cholestasis in preterm infants, with the possibility to add controlled studies on the use of antiviral drugs, especially since all the patients who died were among those who were CMV positive.

We conclude that CMV DNA can be detected in two out of three preterm infants with cholestasis, which is a much higher rate than in age-matched controls. Samples from several locations may be needed to detect viral DNA. Cholestasis with simultaneous detection of CMV DNA seems to be associated with a more severe outcome.

## Data Availability Statement

The raw data will be made available on reasonable request, not without undue reservation.

## Ethics Statement

The studies involving human participants were reviewed and approved by the Stockholm Regional Ethics Committee (reference number 2007/1255-31/3). Written informed consent to participate in this study was provided by the participants' legal guardian/next of kin.

## Author Contributions

JT participated in including cholestatic infants with acquisition of samples, collection of clinical data regarding cases and controls, data management and analysis, interpreting results, and was responsible for manuscript drafts. AE participated in including controls with acquisition of samples, collection of clinical data regarding cases and controls, participated in data management and analysis, and critically revising the manuscript. SO participated in designing the study, performed the major work in including cases, acquiring samples, and performing the laboratory analyses. GA participated in including controls, acquiring samples, collecting clinical data, and critically revising the manuscript. MV and KB participated in designing the study, supervising data collection, interpreting results, and critically revising the manuscript. AN participated in designing the study, interpreting results, and critically revising the manuscript. AR participated in designing the study, performing, supervising and verifying laboratory analyses, interpreting the results, and critically revising the manuscript. BF initiated the study, participated in designing the study, supervising collection of clinical data, interpreting the results, and critically revising the manuscript. CS-N initiated the study, participated in designing the study, supervising the laboratory analyses, interpreting results, and critically revising the manuscript. All authors contributed to the article and approved the submitted version.

## Dedication

SO was the main initiative taker to this study and performed a major part of the organization of clinical sampling collections, laboratory work, and administration for ethical approval of the study. During the project time, she became sick, and was unable to complete the work, and she sadly passed away in the process of manuscript writing of this article. We miss a very dear co-worker and friend, who daily spread joy, warmth, and optimism and showed special empathy and care for those around her. She was a highly competent pediatric rheumatologist, who is also missed by numerous patients that she cared for. We publish this article in her honor and with her dear family left behind her in our thoughts.

## Funding

The Samaritan Foundation, the Swedish Order of Freemasons, the regional agreement between Karolinska Institutet and Region Stockholm (ALF) and strategic funds from Södertälje Hospital granted financial support to JT. The Mjölkdroppen Foundation and the Pediatric Research Foundation of Astrid Lindgren's Children's Hospital granted financial support to SO, MV, and GA. The Child Welfare Society in Stockholm granted financial support to SO. MV received financial support from KI Grants. None of the funders played any role in the design or conduct of the study. All funds were used to financially support leave from regular employment for research.

## Conflict of Interest

The authors declare that the research was conducted in the absence of any commercial or financial relationships that could be construed as a potential conflict of interest.

## Publisher's Note

All claims expressed in this article are solely those of the authors and do not necessarily represent those of their affiliated organizations, or those of the publisher, the editors and the reviewers. Any product that may be evaluated in this article, or claim that may be made by its manufacturer, is not guaranteed or endorsed by the publisher.

## Abbreviations

CMV: cytomegalovirus
cCMV: congenital CMV infection
pCMV: postnatal CMV infection
NEC: necrotizing enterocolitis
DNA: deoxyribonucleic acid
IgG: immunoglobulin G
IgM: immunoglobulin M
CMV-IE: cytomegalovirus immediate early DNA
PCR: polymerase chain reaction
qPCR: quantitative PCR
CT: cycle threshold
SNQ: Swedish neonatal quality register
IVH: intraventricular hemorrhage
ROP: retinopathy of prematurity
BPD: bronchopulmonary dysplasia.

## References

[1] Manicklal S Emery VC Lazzarotto T Boppana SB Gupta RK. The “silent” global burden of congenital cytomegalovirus. Clin Microbiol Rev. (2013) 26:86–102. 10.1128/CMR.00062-12 23297260 PMC3553672

[2] Townsend CL Forsgren M Ahlfors K Ivarsson SA Tookey PA Peckham CS. Long-term outcomes of congenital cytomegalovirus infection in Sweden and the United Kingdom. Clin Infect Dis. (2013) 56:1232–9. 10.1093/cid/cit018 23334811 PMC3616516

[3] Kenneson A Cannon MJ. Review and meta-analysis of the epidemiology of congenital cytomegalovirus (CMV) infection. Rev Med Virol. (2007) 17:253–76. 10.1002/rmv.535 17579921

[4] Kadambari S Pollard AJ Goldacre MJ Goldacre R. Congenital viral infections in England over five decades: a population-based observational study. Lancet Infect Dis. (2020) 20:220–9. 10.1016/S1473-3099(19)30416-5 31708420

[5] Park HW Cho MH Bae SH Lee R Kim KS. Incidence of postnatal CMV infection among breastfed preterm infants: a systematic review and meta-analysis. J Korean Med Sci. (2021) 36:e84. 10.3346/jkms.2021.36.e84 33783146 PMC8007418

[6] Ziemann M Thiele T. Transfusion-transmitted CMV infection - current knowledge and future perspectives. Transfus Med. (2017) 27:238–48. 10.1111/tme.12437 28643867

[7] Kelly MS Benjamin DK Puopolo KM Laughon MM Clark RH Mukhopadhyay S. Postnatal cytomegalovirus infection and the risk for bronchopulmonary dysplasia. JAMA Pediatr. (2015) 169:e153785. 10.1001/jamapediatrics.2015.3785 26642118 PMC4699399

[8] Omarsdottir S Agnarsdottir M Casper C Orrego A Vanpée M Rahbar A. High prevalence of cytomegalovirus infection in surgical intestinal specimens from infants with necrotizing enterocolitis and spontaneous intestinal perforation: a retrospective observational study. J Clin Virol. (2017) 93:57–64. 10.1016/j.jcv.2017.05.022 28633098

[9] Dreher AM Arora N Fowler KB Novak Z Britt WJ Boppana SB. Spectrum of disease and outcome in children with symptomatic congenital cytomegalovirus infection. J Pediatr. (2014) 164:855–9. 10.1016/j.jpeds.2013.12.007 24433826 PMC3982912

[10] Bilavsky E Schwarz M Bar-Sever Z Pardo J Amir J. Hepatic involvement in congenital cytomegalovirus infection - infrequent yet significant. J Viral Hepat. (2015) 22:763–8. 10.1111/jvh.12374 25496231

[11] Fawaz R Baumann U Ekong U Fischler B Hadzic N Mack CL. Guideline for the evaluation of cholestatic jaundice in infants: joint recommendations of the North American Society for pediatric gastroenterology, hepatology, and nutrition and the European Society for pediatric gastroenterology, hepatology, and nutrition. J Pediatr Gastroenterol Nutr. (2017) 64:154–68. 10.1097/MPG.0000000000001334 27429428

[12] Fischler B Ehrnst A Forsgren M Orvell C Nemeth A. The viral association of neonatal cholestasis in Sweden: a possible link between cytomegalovirus infection and extrahepatic biliary atresia. J Pediatr Gastroenterol Nutr. (1998) 27:57–64. 10.1097/00005176-199807000-00010 9669727

[13] Chatterjee A Mukherjee S Basu B Roy D Basu R Ghosh H. Insight into the distinctive paradigm of human cytomegalovirus associated intrahepatic and extrahepatic cholestasis in neonates. Sci Rep. (2020) 10:15861. 10.1038/s41598-020-73009-z 32985571 PMC7522230

[14] Gottesman LE Del Vecchio MT Aronoff SC. Etiologies of conjugated hyperbilirubinemia in infancy: a systematic review of 1692 subjects. BMC Pediatr. (2015) 15:192. 10.1186/s12887-015-0506-5 26589959 PMC4654877

[15] Meshram H Velhal S Padwal V Sutar J Kadam R Pereira J. Hepatic interferon γ and tumor necrosis factor a expression in infants with neonatal cholestasis and cytomegalovirus infection. Clin Exp Hepatol. (2020) 6:367–73. 10.5114/ceh.2020.102172 33511286 PMC7816637

[16] Goel A Chaudhari S Sutar J Bhonde G Bhatnagar S Patel V. Detection of cytomegalovirus in liver tissue by polymerase chain reaction in infants with neonatal cholestasis. Pediatr Infect Dis J. (2018) 37:632–6. 10.1097/INF.0000000000001889 29389827

[17] Hojsak I Colomb V Braegger C Bronsky J Campoy C Domellof M. ESPGHAN Committee on nutrition position paper. Intravenous lipid emulsions and risk of hepatotoxicity in infants and children: a systematic review and meta-analysis. J Pediatr Gastroenterol Nutr. (2016) 62:776–92. 10.1097/MPG.0000000000001121 26825766

[18] Karila K Anttila A Iber T Pakarinen M Koivusalo A. Intestinal failure associated cholestasis in surgical necrotizing enterocolitis and spontaneous intestinal perforation. J Pediatr Surg. (2019) 54:460–4. 10.1016/j.jpedsurg.2018.10.043 30413273

[19] Teng J Arnell H Bohlin K Nemeth A Fischler B. Impact of parenteral fat composition on cholestasis in preterm infants. J Pediatr Gastroenterol Nutr. (2015) 60:702–7. 10.1097/MPG.0000000000000739 25633496

[20] Neuberger P Hamprecht K Vochem M Maschmann J Speer CP Jahn G. Case-control study of symptoms and neonatal outcome of human milk-transmitted cytomegalovirus infection in premature infants. J Pediatr. (2006) 148:326–31. 10.1016/j.jpeds.2005.09.030 16615961

[21] Tufano M Nicastro E Giliberti P Vegnente A Raimondi F Iorio R. Cholestasis in neonatal intensive care unit: incidence, aetiology and management. Acta Paediatr. (2009) 98:1756–61. 10.1111/j.1651-2227.2009.01464.x 19664101

[22] Lee HH Jung JM Nam SH Lim G Chung ML. Risk factor analysis of parenteral nutrition-associated cholestasis in extremely low birth weight infants. Acta Paediatr. (2016) 105:e313–9. 10.1111/apa.13441 27097151

[23] Teng J Bohlin K Nemeth A Fischler B. Cholestasis after very preterm birth was associated with adverse neonatal outcomes but no significant long-term liver disease: a population-based study. Acta Paediatr. (2021) 110:141–8. 10.1111/apa.15408 32524628

[24] Xu X Estekizadeh A Davoudi B Varani S Malmström V Rahbar A. Detection of human cytomegalovirus in synovial neutrophils obtained from patients with rheumatoid arthritis. Scand J Rheumatol. (2020) 50:1–6. 10.1080/03009742.2020.1825798 33243069

[25] Bowerman RA Donn SM Silver TM Jaffe MH. Natural history of neonatal periventricular/intraventricular hemorrhage and its complications: sonographic observations. AJR Am J Roentgenol. (1984) 143:1041–52. 10.2214/ajr.143.5.1041 6385669

[26] Bell MJ Ternberg JL Feigin RD Keating JP Marshall R Barton L. Neonatal necrotizing enterocolitis. Therapeutic decisions based upon clinical staging. Ann Surg. (1978) 187:1–7. 10.1097/00000658-197801000-00001 413500 PMC1396409

[27] International Committee for the Classification of Retinopathy of Prematurity. The international classification of retinopathy of prematurity revisited. Arch Ophthalmol. (2005) 123:991-9. 10.1001/archopht.123.7.991 16009843

[28] Ehrenkranz RA Walsh MC Vohr BR Jobe AH Wright LL Fanaroff AA. Validation of the National Institutes of Health consensus definition of bronchopulmonary dysplasia. Pediatrics. (2005) 116:1353–60. 10.1542/peds.2005-0249 16322158

[29] Fischler B Rodensjö P Nemeth A Forsgren M Lewensohn-Fuchs I. Cytomegalovirus DNA detection on guthrie cards in patients with neonatal cholestasis. Arch Dis Child Fetal Neonatal Ed. (1999) 80:F130–4. 10.1136/fn.80.2.F130 10325791 PMC1720908

[30] Zuhair M Smit GSA Wallis G Jabbar F Smith C Devleesschauwer B. Estimation of the worldwide seroprevalence of cytomegalovirus: a systematic review and meta-analysis. Rev Med Virol. (2019) 29:e2034. 10.1002/rmv.2034 30706584

[31] Choi R Lee S Lee SG Lee EH. Seroprevalence of CMV IgG and IgM in Korean women of childbearing age. J Clin Lab Anal. (2021) 35:e23716. 10.1002/jcla.23716 33783845 PMC8059731

[32] Odland ML Strand KM Nordbø SA Forsmo S Austgulen R Iversen AC. Changing patterns of cytomegalovirus seroprevalence among pregnant women in Norway between 1995 and 2009 examined in the Norwegian Mother and Child cohort study and two cohorts from Sor-Trondelag County: a cross-sectional study. BMJ Open. (2013) 3:e003066. 10.1136/bmjopen-2013-003066 24078749 PMC3787407

[33] Söderberg-Nauclér C Fish KN Nelson JA. Reactivation of latent human cytomegalovirus by allogeneic stimulation of blood cells from healthy donors. Cell. (1997) 91:119–26. 10.1016/S0092-8674(01)80014-3 9335340

