# Supplementary figures and images for: High Rate of Cytomegalovirus Detection in Cholestatic Preterm Infants
Source: Front Pediatr. 2021 Nov 24;9:754941. doi: 10.3389/fped.2021.754941 (PMC8652112; doi:10.3389/fped.2021.754941)

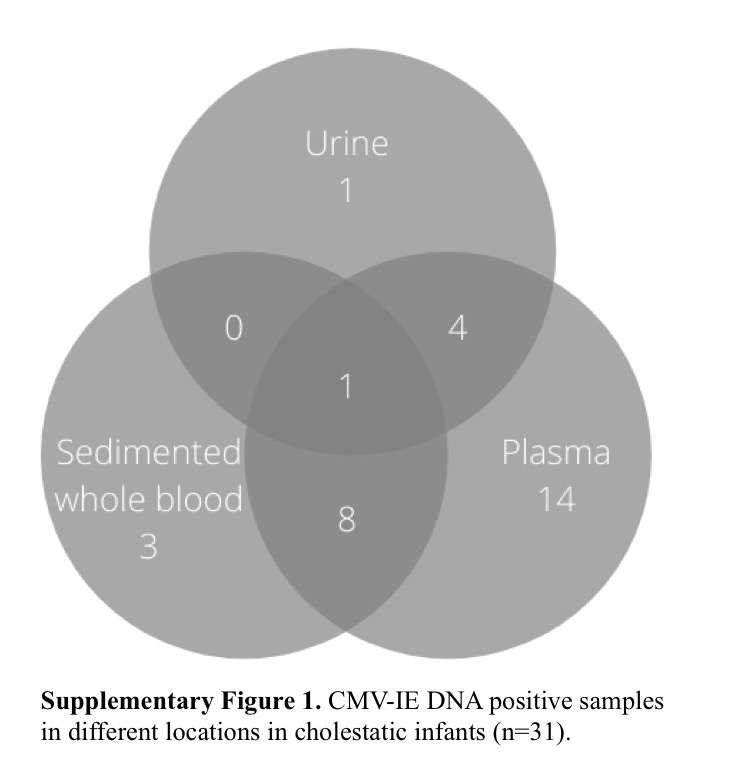

Supplement: Supplementary file 1 [file Image_1.JPEG]
